# Ficolins do not alter host immune responses to lipopolysaccharide-induced inflammation *in vivo*

**DOI:** 10.1038/s41598-017-04121-w

**Published:** 2017-06-20

**Authors:** Ninette Genster, Olga Østrup, Camilla Schjalm, Tom Eirik Mollnes, Jack B. Cowland, Peter Garred

**Affiliations:** 1 Laboratory of Molecular Medicine, Department of Clinical Immunology, Section 7631, Rigshospitalet, Faculty of Health and Medical Sciences, University of Copenhagen, Copenhagen, Denmark; 2 Center for Genomic Medicine, Rigshospitalet, Copenhagen University Hospital, Blegdamsvej 9, DK-2100 Copenhagen, Denmark; 3 Department of Immunology, Oslo University Hospital, Rikshospitalet, 0424 Oslo, Norway; 4 Research Laboratory, Nordland Hospital, Bodø, and K.J. Jebsen TREC, University of Tromsø, Tromsø, Norway; 5 Centre of Molecular Inflammation Research, Norwegian University of Science and Technology, Trondheim, Norway; 6 The Granulocyte Research Laboratory, Department of Hematology, Copenhagen University Hospital, Copenhagen, Denmark; 7 Department of Clinical Genetics, Copenhagen University Hospital, Copenhagen, Denmark

## Abstract

Ficolins are a family of pattern recognition molecules that are capable of activating the lectin pathway of complement. A limited number of reports have demonstrated a protective role of ficolins in animal models of infection. In addition, an immune modulatory role of ficolins has been suggested. Yet, the contribution of ficolins to inflammatory disease processes remains elusive. To address this, we investigated ficolin deficient mice during a lipopolysaccharide (LPS)-induced model of systemic inflammation. Although murine serum ficolin was shown to bind LPS *in vitro*, there was no difference between wildtype and ficolin deficient mice in morbidity and mortality by LPS-induced inflammation. Moreover, there was no difference between wildtype and ficolin deficient mice in the inflammatory cytokine profiles after LPS challenge. These findings were substantiated by microarray analysis revealing an unaltered spleen transcriptome profile in ficolin deficient mice compared to wildtype mice. Collectively, results from this study demonstrate that ficolins are not involved in host response to LPS-induced systemic inflammation.

## Introduction

The ficolins are a family of pattern recognition molecules that initiate the lectin pathway of complement^1–3^. Humans express three ficolins: ficolin-1(M-ficolin)^4, 5^ in bone marrow and myeloid cells, ficolin-2(L-ficolin)^6^ in liver and ficolin-3(H-ficolin)^7^ in liver and lungs. All the human ficolins are present in serum^8–10^. Mice express two ficolins: ficolin-A^11^ in liver and spleen and ficolin-B^12^ in bone marrow. Both proteins are present in serum, although ficolin-B only in very low concentration^13^. Ficolin-B is the homologue of human ficolin-1, ficolin-A is closely related to human ficolin-2 whereas a third ficolin gene exists as a pseudogene in mice^14^. The ficolins recognize pathogen-associated molecular patterns on the surface of microorganisms^15–19^ and DNA on apoptotic and necrotic host cells^20, 21^. Upon pathogen recognition, the ficolin associated serine proteases, named MASPs, catalyze cleavage of complement components C4 and C2, leading to C3 convertase (C4b2a) formation^22^. The C3 convertase cleaves C3 into the opsonin C3b and the anaphylatoxin C3a. Activation of C3 also leads to downstream formation of the C5 convertase (C4b2a3b) which cleaves C5 into the anaphylatoxin C5a and the fragment C5b. C5b initiates formation of the lytic terminal membrane attack complex (C5b-9). In this way, effector functions of the complement cascade, i.e. opsonization, lysis and generation of an inflammatory response, result in elimination of the target. Therefore, complement and ficolins generally have a host-protective effect.

In certain situations, however, an exaggerated activation of the complement cascade can have detrimental effects due to activation of inflammatory cells and secretion of pro-inflammatory cytokines^23, 24^. Sepsis is a systemic overwhelming inflammatory response, resulting from uncontrolled bacterial spread during infection, which can lead to tissue damage, multiple organ failure and death^25^. Because this uncontrolled production of pro-inflammatory cytokines can be more damaging than the primary infection, it is important to attenuate this excessive reaction. Lipopolysaccharide (LPS) from the outer membrane of Gram-negative bacteria plays a major role in activating the inflammatory response during sepsis, and administration of LPS in animal models has been widely used to study the inflammatory response to Gram-negative bacteria^26^.

While a protective effect of ficolins has been demonstrated in clinical studies^27–29^ and in mouse models of infection^13, 30–32^, the contribution of ficolins to inflammatory processes is not well-characterized. However, we recently observed that ficolin deficient mice showed decreased production of pro-inflammatory cytokines during local fungal lung infection *in vivo* ^33^. Others have reported that ficolin-2 promotes production of pro-inflammatory cytokines in mouse macrophages *in vitro*, and that ficolin-A promotes TNF and IL-17 production in response to LPS *in vivo* ^32^.

To elaborate on these previous discoveries and to better understand the role of ficolins during inflammation, we subjected ficolin deficient mice to LPS-induced inflammation and evaluated their inflammatory response and survival.

## Results

### Ficolin-A binds to LPS *in vitro*

**Figure 1:**
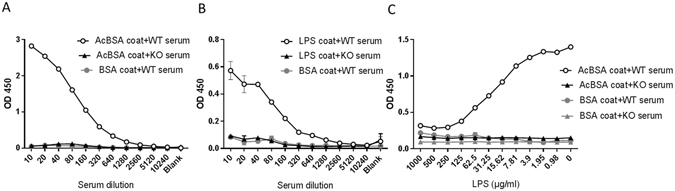
Serum ficolin-A binds to LPS *in vitro*. ELISA plates were coated with of BSA (negative control), the ficolin-A ligand AcBSA (positive control) or LPS (all 5 µg/ml). In **A** and **B**, serial dilutions of mouse serum (WT or ficolin KO) were added to the plates and ficolin-A was detected with an anti-ficolin-A antibody. In **C**, mouse serum (WT or ficolin KO) was diluted 1:50 and pre-incubated with serial dilutions of LPS for 2 h. Subsequently, the samples were added to plates coated with AcBSA or BSA and ficolin-A was detected with an anti-ficolin-A antibody. All graphs show mean ± SEM of duplicate wells and are representative of three independent experiments.

To determine whether ficolin-A binds to LPS, ELISA plates were coated with LPS (from *Escherichia coli* 0111:B4) and serial dilutions of wildtype (WT) or ficolin knockout (KO) serum were added and ficolin-A binding to LPS was detected with an anti-ficolin-A antibody. Plates coated with the ficolin-A ligand acetylated bovine serum albumin (AcBSA) served as a positive control while plates coated with BSA served as a negative control. The results showed a dose-dependent binding of ficolin-A from WT serum to AcBSA and LPS (Fig. 1A,B). As expected, no binding from ficolin KO serum to either of the coats was observed. In another experiment, mouse serum (WT or ficolin KO) was diluted 1:50 and pre-incubated with serial dilutions of LPS for 2 h. When the samples were added to plates coated with AcBSA and ficolin-A was detected with anti-ficolin-A antibody, a dose-dependent inhibition by LPS of ficolin-A binding to its known ligand AcBSA was revealed. These results demonstrated that ficolin-A from mouse serum binds to the LPS used for the subsequent *in vivo* experiments.

### Ficolin deficiency does not impact morbidity and mortality by LPS-induced inflammation

**Figure 2:**
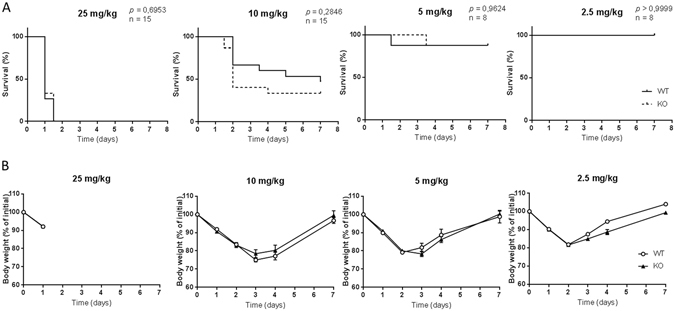
Survival and body weight change is similar in WT and ficolin KO mice after LPS challenge. WT and ficolin KO mice were intraperitoneally injected with LPS of indicated doses and monitored for survival (**A**) and body weight change (**B**) for 7 days. Results were obtained from one (5 mg/kg and 2.5 mg/kg; n = 8) or two (25 mg/kg and 10 mg/kg; n = 15) experiments. In A, Kaplan–Meier survival curves were compared using the log-rank test. In B, graphs show mean ± SEM.

In order to assess the role of ficolins during LPS-induced inflammation *in vivo*, groups of WT mice and ficolin KO mice were injected intraperitoneally (i.p.) with four different doses of LPS and survival time and body weight change was monitored for seven days (Fig. 2). The clinical scores of the animals are presented in Supplementary Fig. S1. Given the highest dose of LPS (25 mg/kg), all mice in WT and ficolin KO groups reached the humane endpoint before day 2 and the median survival time of both groups was 1 day (Fig. 2A). Given a dose of 10 mg/kg, survival rate of WT mice was 47% and of ficolin KO mice 33% and their median survival times were 7 and 2 days, respectively. The difference in survival rates was not statistically significant. After receiving a dose of 5 mg/kg, the survival rate of both WT and ficolin KO groups was 87% and the median survival time was undefined. Given a dose of 2.5 mg/kg, all animals in both groups survived and their median survival time was undefined.

Consistent with the survival recordings, there was no difference in weight loss between WT and ficolin KO mice (Fig. 2B). The clinical scores (Supplementary Fig. S1) and the body weight changes (Fig. 2B) revealed that the mice were affected by the two low doses of LPS although most of them survived the LPS challenge. In addition, control groups of WT and ficolin KO mice received 100 µl of PBS i.p.: their survival was 100% and their body weight remained the same during the 7 day period (Supplementary Fig. S2).

### Ficolin expression is unaltered by LPS-induced inflammation

**Figure 3:**
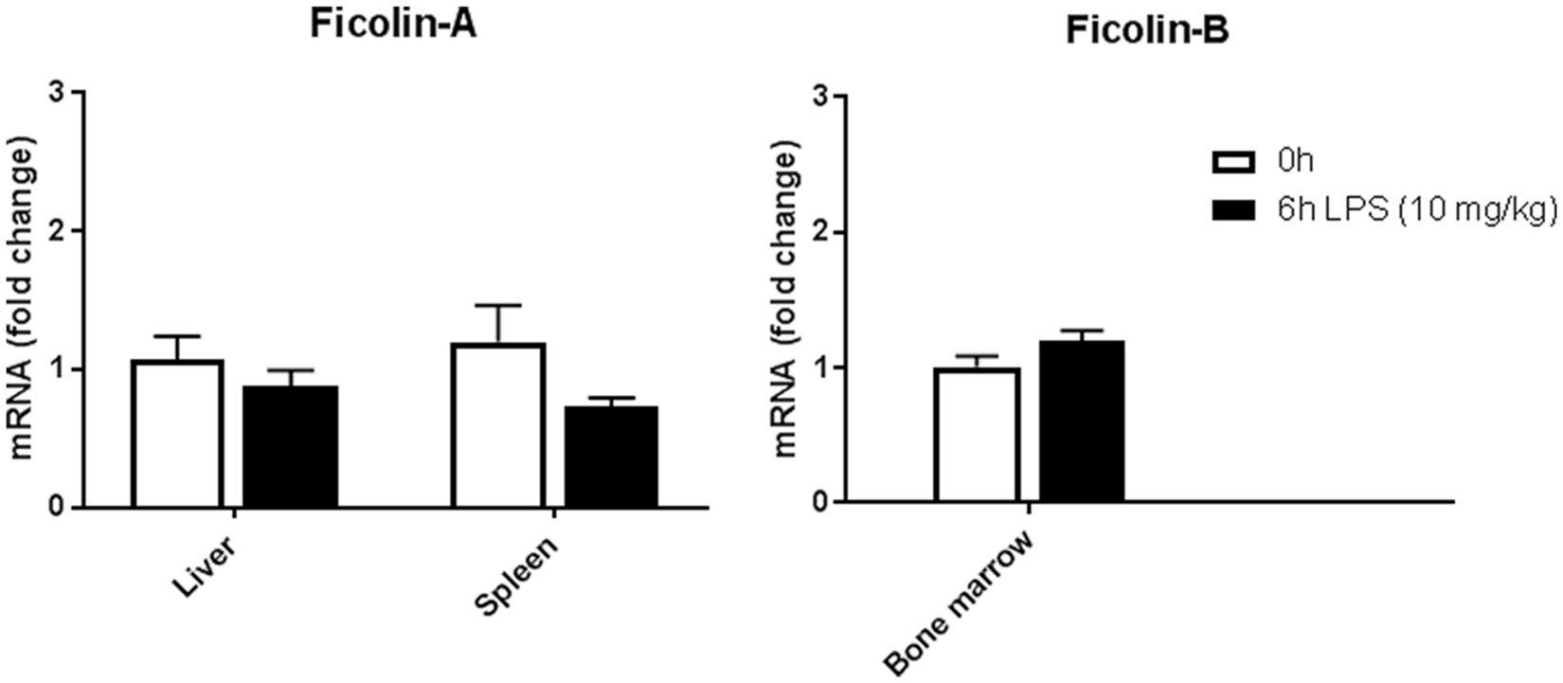
Ficolin expression is unaltered in mice after LPS challenge. Groups of WT mice were untreated or intraperitoneally injected with LPS of a lethal dose (10 mg/kg) and sacrificed after 6 h. Liver, spleen and bone marrow were removed and expression of ficolin-A and ficolin-B in tissue homogenates was measured by qPCR. Data was obtained from one experiment and represent mean ± SEM (n = 5) as the fold change from naive WT mice.

Next, we assessed the expression of ficolins 6 h after LPS challenge of a lethal dose (10 mg/kg). qPCR analysis showed a fold-change in gene expression of ficolin-A in liver of 0.9 and in spleen of 0.7 and of ficolin-B in bone marrow of 1.2 after LPS challenge (Fig. 3); none of these changes were statistically significant.

### Ficolin deficiency does not impact the cytokine profile in response to LPS-induced inflammation

**Figure 4:**
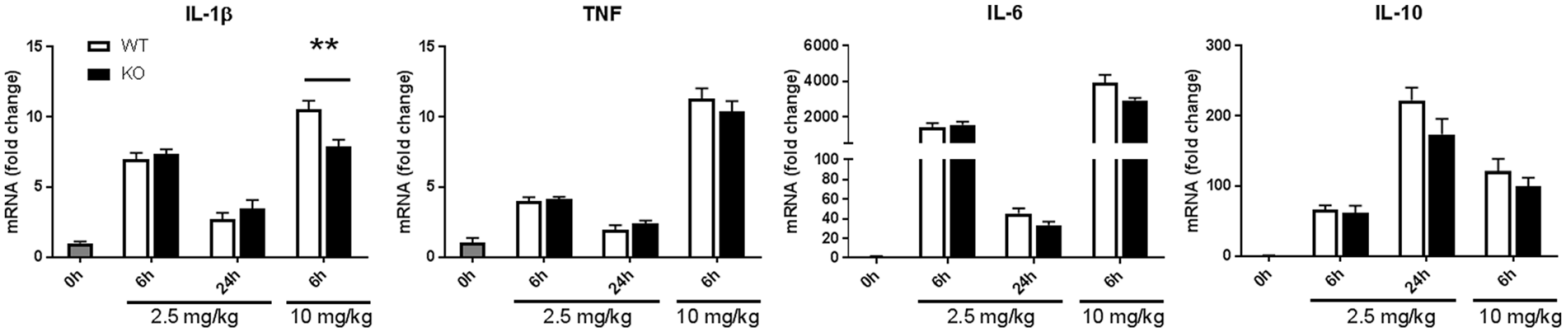
Cyokine expression in spleens of WT and ficolin KO mice after LPS challenge. Groups of WT and ficolin KO mice were intraperitoneally injected with LPS of a sublethal (2.5 mg/kg) or lethal (10 mg/kg) dose and sacrificed at indicated time points. Spleens were removed and expression of inflammatory cytokines in spleen homogenates was measured by qPCR. Data represent mean ± SEM as the fold increase over the control group (naive WT mice); n = 6–8 mice in LPS groups and n = 2 mice in the control group. **p < 0.01 for WT to ficolin KO comparisons (unpaired two-tailed Student’s t test).

**Figure 5:**
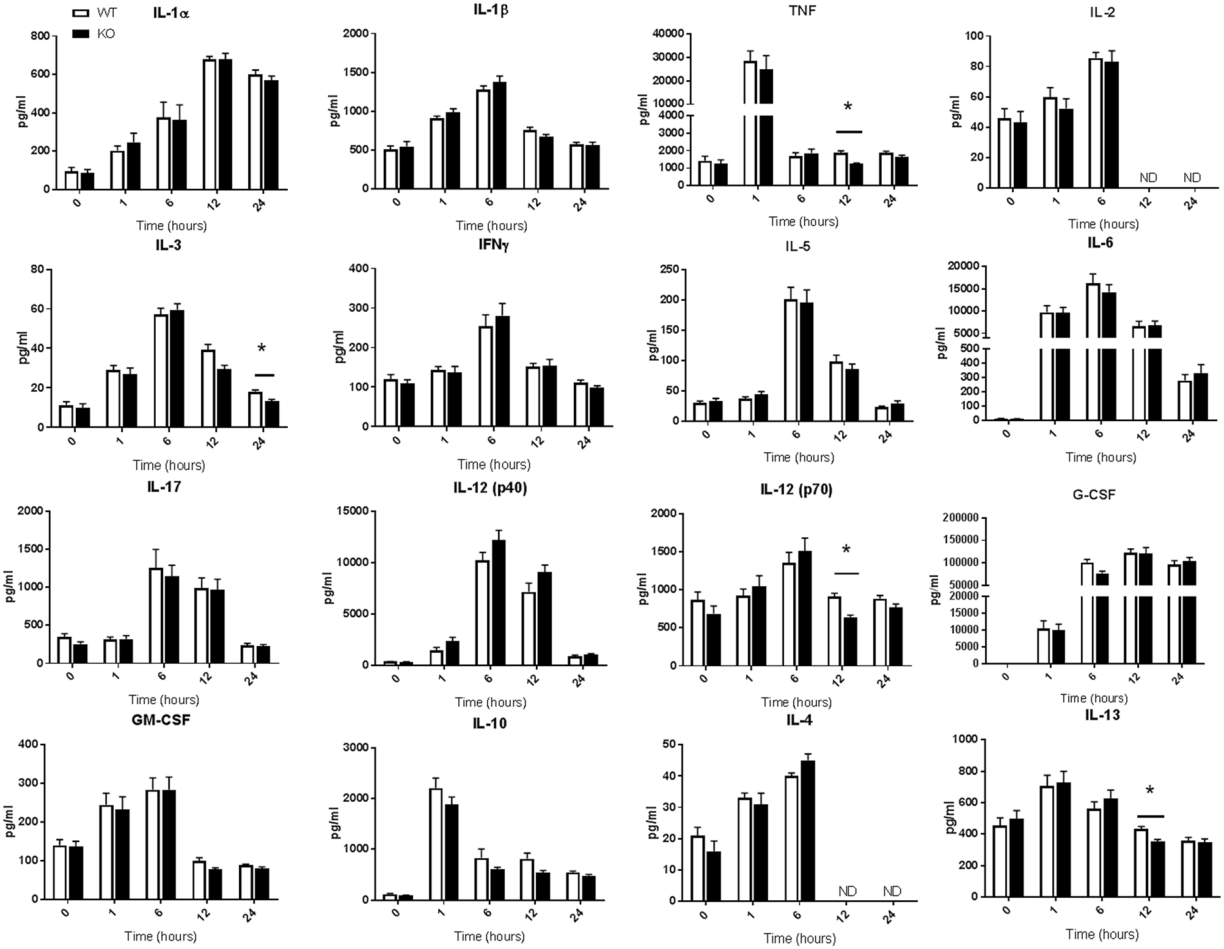
Serum cytokine levels in WT and ficolin KO mice after LPS challenge with a sublethal dose. Groups of WT and ficolin KO mice were intraperitoneally injected with LPS of a sublethal dose (2.5 mg/kg). At indicated time points, blood was collected before the mouse was sacrificed. The serum concentration of inflammatory cytokines was measured by a multiplex Luminex assay; the data was obtained from two independent experiments per time point and represent mean ± SEM (n = 11 mice per group per time point). *p < 0.05 for WT to ficolin KO comparisons (unpaired two-tailed Student’s t test). ND = not determined.

**Figure 6:**
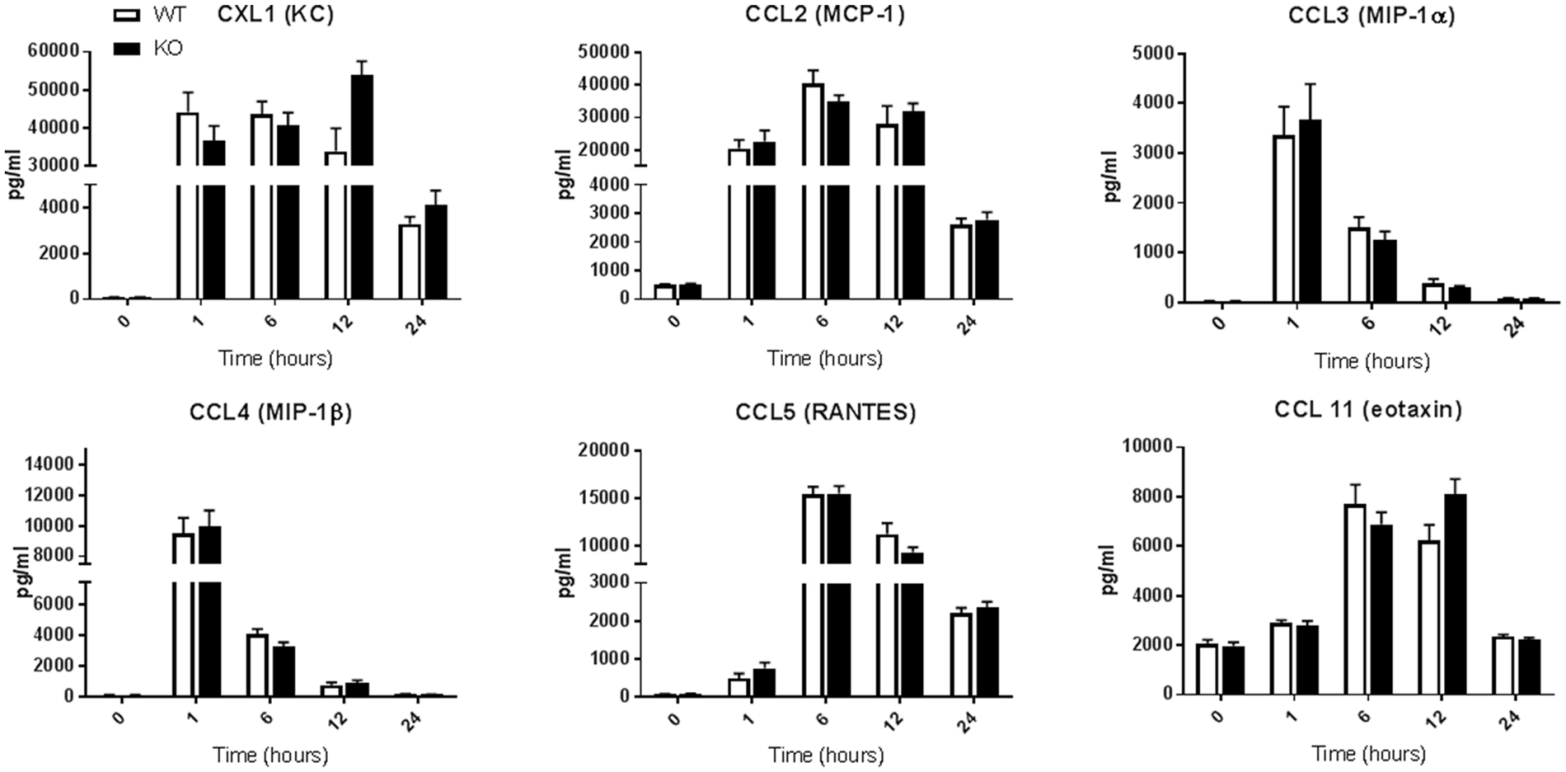
Serum chemokine levels in WT and ficolin KO mice after LPS challenge with a sublethal dose. Groups of WT and ficolin KO mice were intraperitoneally injected with LPS of a sublethal dose (2.5 mg/kg). At indicated time points, blood was collected before the mouse was sacrificed. The serum concentration of chemokines was measured by a multiplex Luminex assay; the data was obtained from two independent experiments per time point and represent mean ± SEM (n = 11 mice per group per time point).

**Figure 7:**
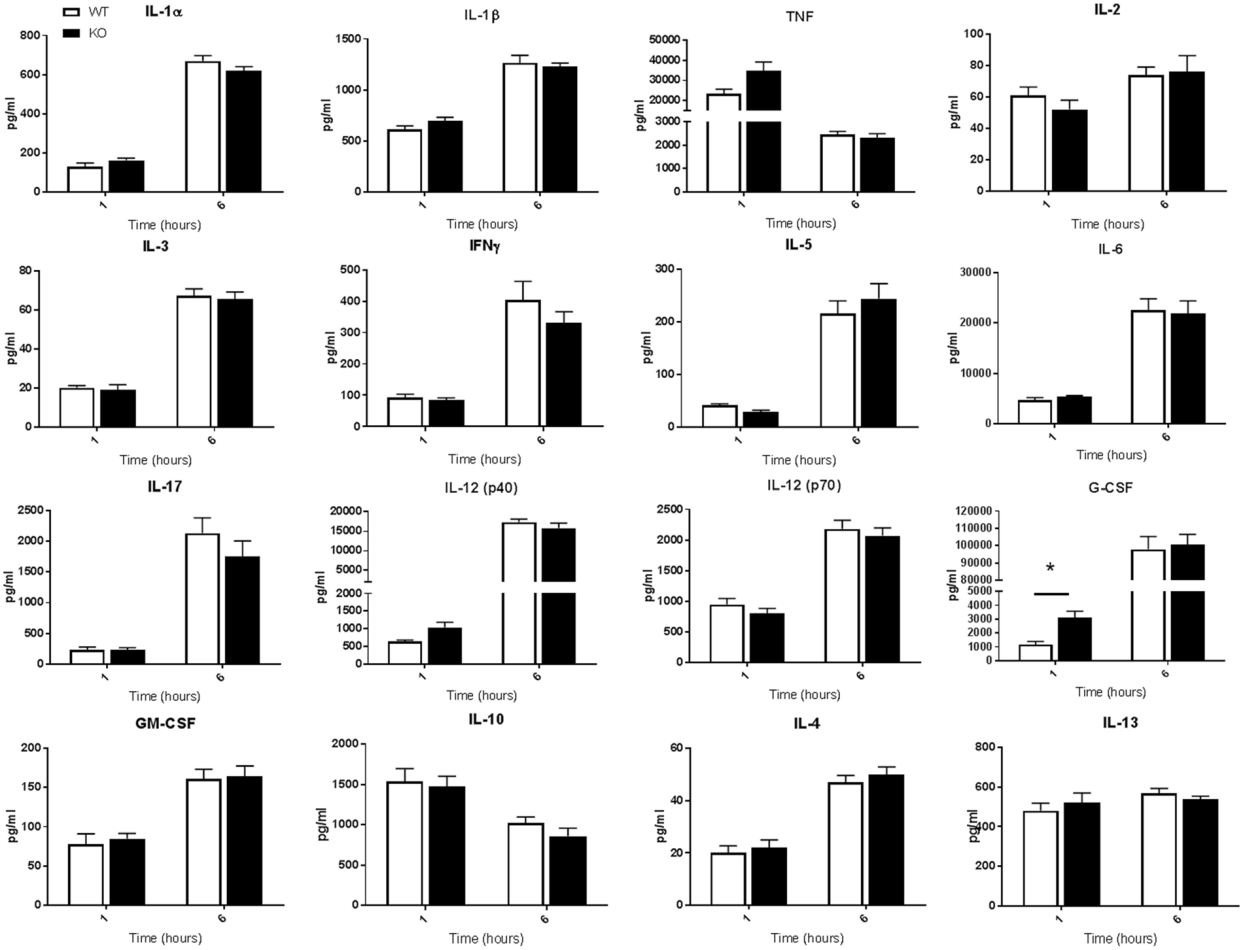
Serum cytokine levels in WT and ficolin KO mice after LPS challenge with a lethal dose. Groups of WT and ficolin KO mice were intraperitoneally injected with LPS of a lethal dose (10 mg/kg). At 1 h or 6 h after challenge, blood was collected before the mouse was sacrificed. The serum concentration of inflammatory cytokines was measured by a multiplex Luminex assay; the data was obtained from two independent experiments per time point and represent mean ± SEM (n = 11 mice per group per time point). *p < 0.05 for WT to ficolin KO comparisons (unpaired two-tailed Student’s t test).

**Figure 8:**
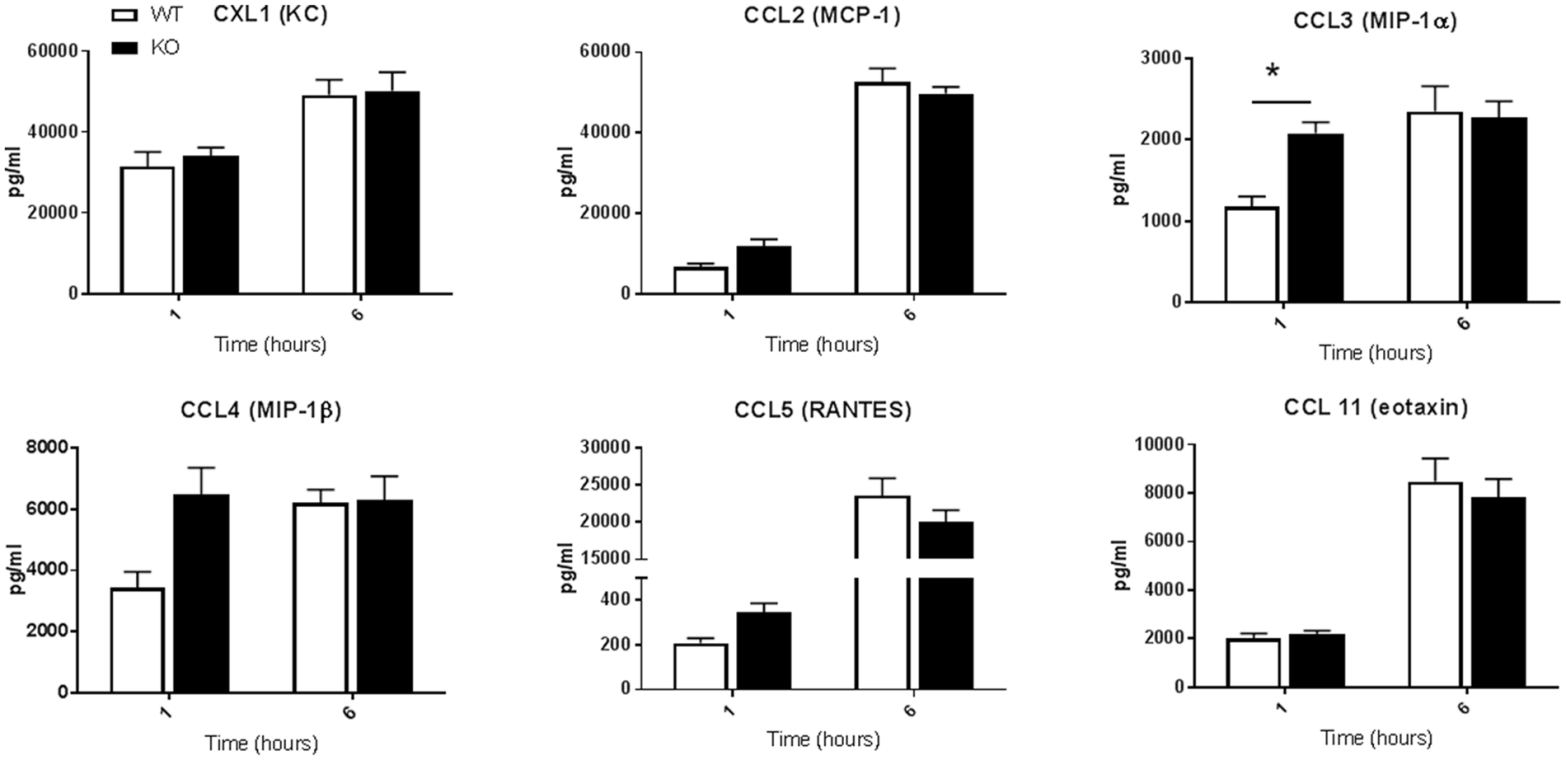
Serum chemokine levels in WT and ficolin KO mice after LPS challenge with a lethal dose. Groups of WT and ficolin KO mice were intraperitoneally injected with LPS of a lethal dose (10 mg/kg). At 1 h or 6 h after challenge, blood was collected before the mouse was sacrificed. The serum concentration of chemokines was measured by a multiplex Luminex assay; the data was obtained from two independent experiments per time point and represent mean ± SEM (n = 11 mice per group per time point). *p < 0.05 for WT to ficolin KO comparisons (unpaired two-tailed Student’s t test).

To evaluate the LPS-induced inflammatory response in the animals, we analyzed the cytokine profiles after LPS challenge (Figs 4–8). First, we determined the gene expression of inflammatory cytokines in spleens of WT and ficolin KO mice (Fig. 4). Groups of mice were either challenged with a lethal dose of LPS (10 mg/kg) and sacrificed after 6 h or challenged with a sublethal dose of LPS (2.5 mg/kg) and sacrificed after 6 h or 24 h, and the mRNA levels of IL-1β, TNF, IL-6 and IL-10 in spleen homogenates were determined. Comparing the expression of the cytokines induced by the two different doses at 6 h, all four cytokines were expressed to a higher extend after the lethal dose. After challenge with a lethal dose of LPS, the expression of all four cytokines were lower at 6 h in ficolin KO mice compared to WT, but only the difference in IL-1β levels was statistically significant. After a sublethal dose of LPS, the pro-inflammatory cytokines (IL-1β, TNF and IL-6) peaked at 6 h, while the anti-inflammatory cytokine IL-10 peaked at 24 h. There was no difference in expression between ficolin KO mice and WT mice challenged with a sublethal dose of LPS.

To further assess the inflammatory response in mice after LPS challenge, we measured the serum levels of 22 inflammatory cytokines and chemokines at various time points after challenge with a sublethal or a lethal dose of LPS (Figs 5–8).

Groups of WT and ficolin KO mice given a sublethal dose of 2.5 mg/kg of LPS i.p. were bled and sacrificed at 0 h, 1 h, 6 h, 12 h or 24 h after LPS challenge (Figs 5,6). Most of the cytokines peaked at 6 h and about half of the cytokines returned to basal level at 24 h. TNF and IL-10 peaked at 1 h whereas IL-1α peaked at 12 h (Fig. 5). Three of the six chemokines peaked early at1h, whereas the other three peaked at 6 h (Fig. 6). The most increased cytokines after LPS challenge included TNF, IL-6, IL-12(p40), G-CSF and the chemokines. Overall, levels of the 22 cytokines and chemokines were similar in WT and ficolin KO mice challenged with a sublethal LPS dose and only levels of a few cytokines were significantly lower in KO mice; TNF at 12 h, IL-3 at 24 h, IL-12(p70) at 12 h and the anti-inflammatory IL-13 at 12 h.

In another set of experiments, groups of WT and ficolin KO mice were given a lethal dose of 10 mg/kg of LPS i.p. and sacrificed at 1 h or 6 h to assess the serum cytokine and chemokine levels (Figs 7, 8). The general cytokine profiles resembled those of mice given a sublethal dose; most of the cytokines peaked at 6 h, whereas TNF and IL-10 peaked at 1 h, and the most increased cytokines were TNF-α, IL-6, IL-12(p40), G-CSF and the chemokines. In accordance to mice given the sublethal dose, the chemokines that peaked after 6 hours were CCL2, CCL5 and CCL11. However, regarding CCL3 and CCL4, the levels clearly declined from 1 h to 6 h in both WT and ficolin KO mice given the sublethal dose (Fig. 6), whereas the levels increased in WT mice and remained the same in KO mice given a lethal dose (Fig. 8). Except for a statistically significant higher level of G-CSF and CCL3 in ficolin KO mice at 1 h, there was no difference in the general pattern of cytokine and chemokine levels between WT and ficolin KO mice given a lethal dose of LPS.

Altogether, these results showed no overall difference between WT and ficolin KO mice in the inflammatory cytokine response after LPS challenge, regardless of the dose given and the time point investigated.

### Ficolin deficiency does not alter the spleen transcriptome profile

**Figure 9:**
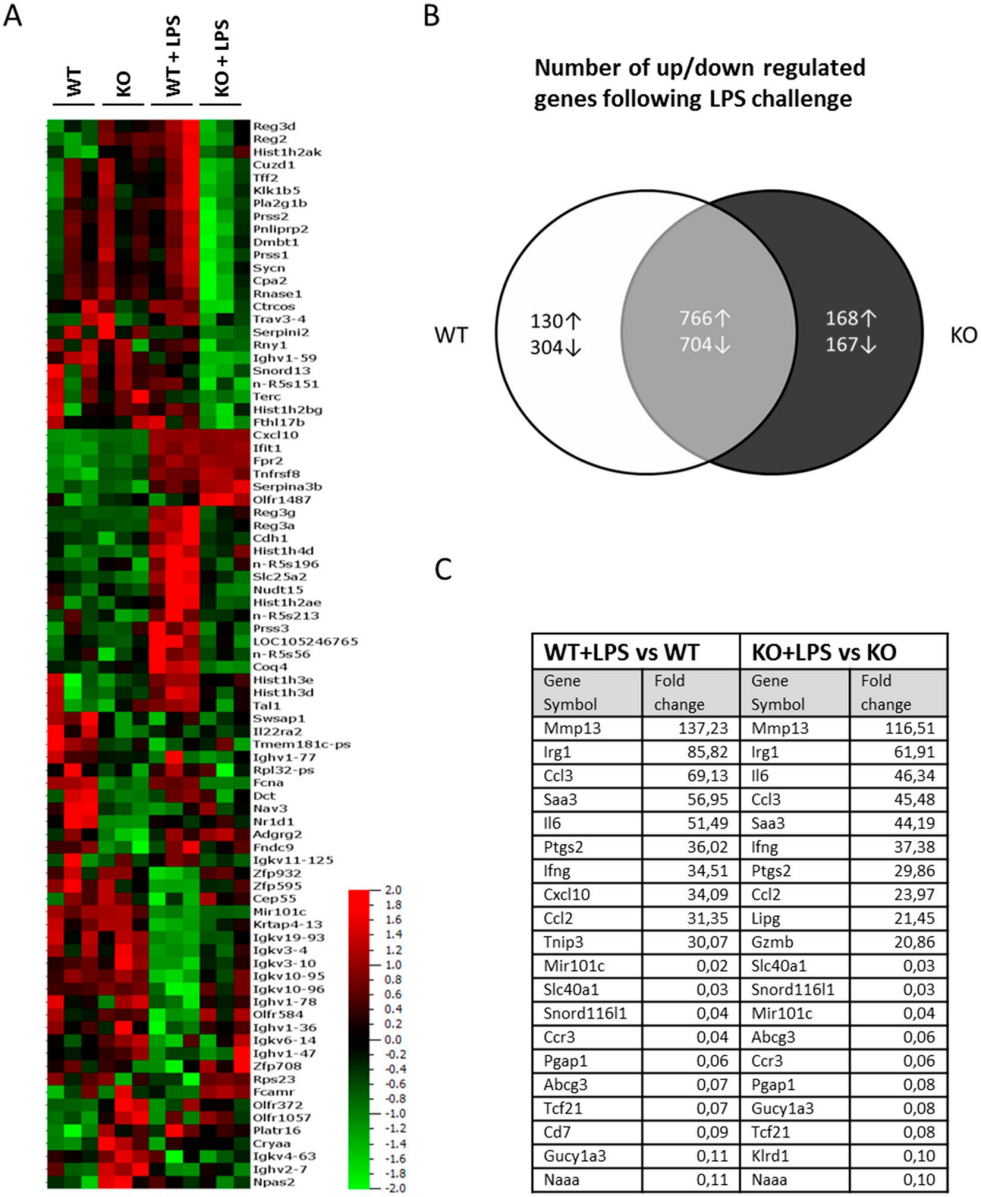
Microarray analysis of differentially expressed genes in WT and ficolin KO mice after LPS challenge. Groups of WT and ficolin KO mice were untreated or intraperitoneally injected with LPS of a lethal dose (10 mg/kg) and sacrificed after 6 h (n = 3 mice per group). Total RNA extracted from spleens was hybridized to the Affymetrix GeneChip Mouse Genome 2.0 ST arrays chip. (**A**) Heatmap showing all experimental samples and 85 genes found as differentially expressed between WT and KO mice in either naive or LPS-challenged state (cut-off: fold-change > 1.5 and p < 0.05). Samples are ordered by the four experimental groups and variables order by hierarchical clustering. (**B**) Venn diagram showing the number of differentially expressed genes induced by LPS challenge (cut-off: fold-change > 2.0 and q < 0.05). The white area contains the number of genes that were only affected in WT mice upon LPS challenge; the black area contains the number of genes that were only affected in ficolin KO mice upon LPS challenge; the grey area contains the number of overlapping genes between WT and ficolin KO mice that were affected upon LPS challenge. (**C**) Table showing the top 20 most changed genes (10 up and 10 downregulated) after LPS challenge in WT and ficolin KO mice (cut-off: fold-change > 2.0 and q < 0.05).

Next, we performed microarray analysis to gain insight to the genome-wide transcriptional changes accompanying ficolin deficiency in spleens from naive or LPS challenged (10 mg/kg for 6 h) mice. Using fold-change > 1.5 and p < 0.05 as criteria for significance (Fig. 9A), we identified 24 differentially expressed genes (DEGs) between ficolin KO and WT mice in the naive state (Supplementary Table S1) and 59 DEGs between ficolin KO mice and WT mice in the LPS challenged state (Supplementary Table S2). If setting the significance border to q < 0.05 only *Fcna* (ficolin-A) would be considered as differentially expressed between KO and WT groups. In general, most of the DEGs did not appear to be related to LPS-induced inflammation. Of note is, however, that *Reg2* (regenerating islet-derived 2) was upregulated in ficolin KO mice (fold-change 7.0) in the naive state, whereas the same gene was downregulated in ficolin KO mice (fold-change 0.1) in the LPS challenged state. We also compared the number of changed genes in ficolin KO mice after LPS challenge to the number of changed genes in WT mice after LPS challenge (Fig. 9B). Using fold-change > 2.0 and q < 0.05 as criteria for significance, we identified 1470 genes that were altered in both WT and ficolin KO mice after LPS challenge. In addition, 434 genes were changed only in WT mice, whereas other 335 genes were changed only in ficolin KO mice. Among the mostly LPS-responsive genes common for ficolin KO and WT mice were chemokines, cytokines and other pro-inflammatory mediators, including *Ccl2*, *Ccl3*, *Ifng*, *Il6*, *Mmp13* (matrix metallopeptidase 13), *Irg1* (Immune Responsive Gene 1), *Saa3* (serum amyloid A 3) and *Ptgs2* (prostaglandin-endoperoxide synthase 2) (Fig. 9C).

Overall, the results show that many genes change in response to LPS in both ficolin KO and WT mice; however, lack of ficolins does not change the transcriptional program in spleens, neither in the naive state nor after LPS challenge. These gene expression data therefore substantiate that ficolin deficiency does not alter the host response to LPS-induced inflammation in mice.

## Discussion

In the present study we show for the first time that ficolins do not contribute to the LPS-induced inflammatory response *in vivo* in mice. The data are consistent using a broad panel of readouts; microarrays and selected qPCRs in tissues in addition to a number of single inflammatory mediators measured in serum. Convincingly, there were no overall differences between ficolin KO mice and WT mice.

Few studies involving ficolin KO mice are found in the literature to date. Endo *et al*. found that survival rates of ficolin-A KO, ficolin-B KO and double KO mice were significantly reduced compared to WT mice after intranasal infection with a *Streptococcus pneumoniae* strain^13^. Reconstitution of ficolin-A improved the survival rate of ficolin-A KO mice, but not of double KO mice, revealing the requirement of both ficolin-A and ficolin-B in the defense against *S. pneumoniae*. Another study also reported a reduced survival rate in ficolin-A KO mice using the same *S. pneumoniae* strain^31^. Reduced survival rates of ficolin-A KO mice compared to WT mice after infection with *influenza A* virus^30^ and *Mycobacterium tuberculosi*s^32^ have also been reported. In addition, we showed that ficolin KO mice showed decreased fungal clearance in the early phase of *A. fumigatus* infection^33^. A common feature of these studies is that they employ lung infection models.

Besides these infection models, ficolin-B KO mice have been investigated in a model of Type1 diabetes related kidney desease^34^, ficolin-A KO mice have been investigated in a model of collagen antibody-induced arthritis^35^ and ficolin-A KO as well as ficolin-B KO mice have been subjected to intravenous injection of mitochondria^36^. There was no difference between ficolin KO mice and WT mice in any of these experimental models. In the present study we found that although serum ficolin-A recognize LPS *in vitro*, ficolin KO mice did not show an altered response to four different doses of LPS *in vivo* compared to WT mice. Hence our model of endotoxemia is featured on the list of non-infectious mouse models in which ficolins seem redundant.

Mannose-binding lectin (MBL) is the most widely studied family member of the lectin pathway complement initiators. In 2003 our research group published that MBL gene polymorphisms, giving rise to low levels of MBL, were associated with the development of sepsis and increased risk of fatal outcome from the disease^37^. Mice express two forms of the molecule: MBL-A and MBL-C. The MBL-A KO mouse has been examined in the cecal ligation and puncture (CLP) model of acute septic peritonitis and interestingly it was revealed that MBL-A KO mice showed increased survival compared to WT mice and to C3 KO mice^38^. While the CLP model induces a polymicrobial sepsis that mimics the later phase of human sepsis, the LPS model, which is used in the present study, induces systemic inflammation that mimics many of the initial clinical features of sepsis, but without bacteremia^26^. It would be interesting to test whether ficolin KO mice have a survival advantage in the CLP model like the MBL-A KO mice. All things considered, it is conceivable that an effect of the ficolins require the presence of a (whole) microbe. Recent observations revealed that consumption of ficolin-2 in patients with necrotizing soft tissue infection was associated with increased risk of fatal outcome^39^. However, necrotizing soft tissue infection is predominantly caused by Gram-positive polymicrobial pathogens such as Group A Streptococcus. Nonetheless, it may underscore the importance of the ficolins in clinical situations involving polymicrobial infections.

An early study found that serum MBL-A levels in mice were increased approximately 2-fold, peaking 32 h after the mice received an LPS-injection, while the MBL-C levels did not increase^40^. A later study found that serum MBL-A and MBL-C levels were reduced for 1 week after CLP, while the mRNA levels where unchanged, indicating consumption rather than decreased protein expression^41^. Another study found that ficolin-B mRNA expression was higher in neutrophils isolated from mice subjected to CLP compared to naive mice^42^. In the present study, we did not detect a change in gene expression of ficolin-A or ficolin-B after LPS challenge. In humans, endobronchial administration of LPS was associated with increased levels of alveolar ficolin-3 and MBL, and intravenous administration of LPS was associated with increased levels of plasma ficolin-1^43^. No change in gene expression of ficolin-1 or ficolin-3 was observed. Interestingly, homozygosity for minor alleles resulting in increased ficolin-1 levels was associated with fatal outcome in patients with systemic inflammation/sepsis^44^. These studies suggest that, at least in humans, the ficolins play a role in the local and systemic host defense in response to LPS and sepsis. It is important to note that although mouse models are regarded as acceptable models of sepsis in humans, there are multiple factors that differ between the two species, including differences in the ficolin molecules and the host response to LPS.

Prior research has demonstrated the ability of ficolins to modulate the cytokine response *in vitro* and *in vivo*. *In vitro*, one study showed that ficolin-2 promoted production of IFN-γ, IL-17A, TNF and IL-6 by murine macrophages^32^, whereas another study showed that ficolin-A (or MBL-A) suppressed the LPS-mediated IL-6 and TNF production by murine mast cells^45^. *In vivo*, we found that mRNA and proteins levels IL-1β, IL-6 and TNF were reduced by approximately 30% in lungs of ficolin KO mice compared to WT mice after *A. fumigatus* infection^33^, whereas others found that mRNA levels of TNF-α, IFN-γ and CLCX2 (MIP-2) was increased in lungs from ficolin-A KO mice compared to WT mice after *S. pneumonia* infection^31^. In another study, TNF and IL-17A serum levels were measured in ficolin-A KO mice and WT mice after intravenous LPS challenge: TNF levels were decreased in the KO mice at 3 h and 6 h after LPS challenge (but not at 10 h) and IL-17A levels were decreased in the KO mice at 3 h (but not at 6 h) after LPS challenge^32^. In the present study there were only a few significantly changed cytokines in ficolin KO mice compared to WT mice, including decreased serum TNF at 12 h after sublethal LPS challenge. However, since none of these cytokines were significantly changed in more than one of the three analyses employed (qPCR, Luminex and microarray), we conclude that there was no overall difference in the cytokine response between ficolin KO mice and WT mice after challenge with a sublethal or a lethal dose of LPS. Collectively, these diverse results from different studies indicate that the ability of the ficolins to modulate cytokine responses is dependent on the underlying disease.

In the present study, whole genome transcriptome analysis confirmed that ficolins were not implicated in LPS-induced inflammation in the animals, and perhaps equally important that there was no significant difference between naive ficolin KO mice and WT mice in spleen expression profiles except, not surprisingly, expression of ficolin-A. Hence, ficolin deficiency per se does not affect transcription in naive mice. Furthermore, the possibility of flanking-allele effects in ficolin KO mice can be ruled out. To our knowledge, this is the first study to provide whole genome transcription profiles of ficolin KO mice.

Despite a growing number of reports on ficolin properties in various *in vitro* and *in vivo* model systems, a clear understanding of the actual biological functions of these conserved and phylogenetically ancient molecules remains elusive. In conclusion this study provided important new information about the ficolins. We examined the inflammatory response to LPS in ficolin KO mice in detail; we assessed the cytokine profiles and performed whole genome transcriptome profiling. The results unequivocally documented that ficolins are not involved in the LPS-induced inflammatory response in mice.

## Methods

### Reagents

LPS (*E. coli* 0111:B4, product no. L 2630 from Sigma-Aldrich) was suspended at a concentration of 5 mg/ml in sterile PBS and stored in aliquots at −20 °C. LPS was further diluted to the working concentration in sterile PBS on the day of use. Maxisorp polystyrene microtiter plates were purchased from NUNC. PBS-buffer (10 mM Na_2_HPO_4_, 1.47 mM KH_2_PO_4_, 137 mM NaCl, 2.7 mM KCl, pH 7.4) and Barbital- buffer (4 mM C_8_H_11_N_2_NaO_3_, 145 mM NaCl, 2.6 mM CaCl_2_, 2.1 mM MgCl_2_, pH 7.4) and sulphuric acid were all acquired from the hospital pharmacy (RegionH Apoteket, Rigshospitalet, Copenhagen, Denmark). Tween-20 was purchased from Merck. Bovine serum albumin (BSA) (A3803) was purchased from Sigma-Aldrich and acetylated (AcBSA) as described previously^46^. Horseradish-peroxidase (HRP) conjugated rabbit-anti-rat IgG was from Dako, Streptavidin-HRP was from GE Healthcare and TMB ONE was from Kem-En-Tec Diagnostics.

### ELISA binding assays

For the ELISA binding assay microtiter plates were coated overnight with 100 µl LPS in1M NaCl, AcBSA (positive control^47^) or BSA (negative control), all 5 µg/ml in PBS. Barbital-Tween buffer was used for all subsequent washing and dilutions. Serial dilutions of mouse serum (WT or KO) were added to the wells and plates were incubated for 2 h at 37 °C. After washing, binding of ficolin-A to the coat was detected with a biotinylated in-house produced rat anti-mouse ficolin-A (1 µg/ml, overnight at 4 °C) followed by washing and Streptavidin-HRP (1:2000, 1 h shaking at room temperature). For the ELISA inhibition assay, mouse serum (WT or KO) was diluted 1:50 in barb-T buffer and pre-incubated with serial dilutions of LPS for 2 h at 37 °C before adding the samples to plates coated with AcBSA or BSA as above. Plates were incubated for 2 h at 37 °C, washed, and binding of ficolin-A to the coat was detected with in-house produced rat anti-mouse ficolin-A (1 µg/ml, overnight at 4 °C) followed by washing and rabbit-anti-rat HRP (1:2000, 1 h shaking at room temperature). All plates were washed and developed for 10–15 min with 100 µl/well TMB-one substrate and the reaction was stopped with 100 µl/well 0.2 M sulphuric acid. The optical density was measured at 450 nm.

### Mice

Mice double deficient of ficolin-A and ficolin-B were generated as previously described^13, 33^. Heterozygous mice were crossed and the resulting offspring were used for homozygous breeding of WT and double KO mice; mice used in this study belong to the F4 generations. Female mice were used at 10–12 weeks of age. The mice were housed in pathogen-free, temperature-controlled and air-conditioned facilities with a 12 h light/dark cycle. Breeding as well as all animal experiments were conducted at the Department of Experimental Medicine, University of Copenhagen, and Rigshospitalet University Hospital, Copenhagen, Denmark. All animal experiments were approved by the Danish Animal Experiments Inspectorate (permission No. 2016-15-0201-00896) and conducted according to the national Animal Experimentation Act (LBK No. 475 from May 15, 2014). The review board at the Faculty of Health and Medical Sciences, University of Copenhagen, approved this study (P16-247).

### LPS challenge

Weight-matched mice (20 ± 2 g) received 100 µl i.p. of the indicated doses of LPS. Control mice in the survival study received 100 µl i.p. of PBS. For the survival studies, the mice were assessed every 2-8 hours (based on severity of illness), weighted each morning, and survival/clinical score was recorded twice a day (8 a.m. and 8 p.m.) for 7days. The severity of the clinical appearance was scored as follows: 0, not affected; 1, slightly affected (mild piloerection and slightly hunched posture, normal activity); 2, affected (hunched posture, mild piloerection, less active); 3, severely affected (very hunched posture and severe piloerection, inactive). Animals were euthanized if they reached a score of 3. For the collection of serum and organs for further analysis, the mice were challenged with indicated doses of LPS and sacrificed by cervical dislocation at indicated time points.

### Quantitative PCR

Mice were challenged with a lethal dose (10 mg/kg) of LPS and sacrificed after 6 h. In other experiments mice were challenged with a sublethal dose (2.5 mg/kg) and sacrificed after 6 h or 24 h. Naive mice served as controls. Organs were removed and stored in RNAlater (Applied Biosystems). Tissue samples were homogenized with a mechanical homogenizer (TissueRuptor, Qiagen) and RNA was extracted using the Maxwell 16 tissue LEV Total RNA Purification Kit (Promega) following the manufacturer’s recommendations. Total RNA was quantified using a Qubit Fluorometer (Invitrogen) and 1 µg of total RNA was reverse transcribed with the MuLV Reverse transcriptase (Applied Biosystems #N808-0018). Cytokine gene expression in spleens was assessed by quantitative real-time PCR with TaqMan probes specific for mouse TBP (mM00446971_m1), IL-1β (Mm0434228_m1), TNF(Mm00443258_m1), IL-6 (Mm00446190_m1) and IL-10 (Mm00439614_m1), obtained from Applied Biosystems. Quantitative Real-time PCR was performed in triplicate loading 50 ng (IL-1 and TNF) or 100 ng (IL-6 and IL-10) of cDNA per reaction and carried out on the Stratagene Mx3005 P Real-Time PCR system (Agilent Technologies). Reaction conditions included incubation at 50 °C for 2 min. and 95 °C for 10 min. followed by 40 cycles at 95 °C for 15 s and 60 °C for 1 min. PCR runs included no-template controls and no-reverse transcriptase controls. The target genes were normalized to the housekeeping gene, TBP, and results were expressed as fold changes from naive WT mice (there was no difference in gene expression between naive WT and KO mice, data not shown) using the delta-delta *Ct* method. Ficolin-A gene expression in liver and spleen and ficolin-B gene expression in bone marrow was assessed under the same conditions as above with TaqMan probes specific for mouse TBP (mM00446971_m1), ficolin-A (Mm00484287_m1) and ficolin-B (Mm01332438_m1) obtained from Applied Biosystems.

### Luminex assay

After the administration of a lethal (10 mg/kg) or sublethal (2.5 mg/kg) dose of LPS, serum was obtained from submandibular blood collection before the animal was sacrificed. Blood was also collected from naive mice. The serum was aliquoted and kept at −80 °C until further analysis. In experiments in which a lethal dose of LPS was given, blood was collected at 1 h or 6 h after LPS challenge in separate experiments. In experiments in which a sublethal dose of LPS was given, blood was collected at 1 h, 6 h, 12 h or 24 h after LPS challenge. Blood was also collected from naive mice (0 h). All serum samples were assayed in duplicate with a 23-plex mouse cytokine kit and read using the Bio-Plex 200 system and the Bio-Plex Manager software version 6.0 according to manufacturer’s protocol (Bio-Rad Laboratories). The profile of the cytokines analyzed was IL-1α, IL-1β, IL-2, IL-3, IL-4, IL-5, IL-6, IL-9, IL-10, IL-12 p40, IL-12 p70, IL-13, IL-17, eotaxin (CCL11), G-CSF, GM-CSF, IFN-γ, KC, MCP-1, MIP-1α, MIP-1β, RANTES and TNF. IL-9 was excluded from the analysis because it was undetectable in all samples. On one plate, containing the 12 h and 24 h samples, IL-2 and IL-4 was also undetectable, for which reason these were excluded.

### Microarray analysis

RNA from spleens of naive mice and mice challenged with LPS was isolated as described above for qPCR. Subsequently, RNA was reverse transcribed and used for cRNA synthesis, labelling and hybridization with GeneChip Mouse Genome 2.0 ST arrays (Affymetrix) according to the manufacturer standard protocol available at www.affymetrix.com. The arrays were washed and stained with phycoerytrin conjugated streptavidin using the Affymetrix Fluidics Station 450, and the arrays were scanned in the Affymetrix GeneArray 3000 7 G scanner to generate fluorescent images. Cell intensity files (.CEL files) were generated in the GeneChip Command Console Software (AGCC; Affymetrix). The microarray data are deposited to Gene Expression Omnibus (accession number GSE95125).

Bioinformatics analysis was performed using Qlucore Omics Explorer 3.2. Raw intensity.CEL files were preprocessed by quantile normalization, and gene summaries were extracted via robust multi-array average (RMA)^48^. By comparison of groups with LPS with groups without LPS (i.e. KO + LPS versus KO-LPS and WT + LPS versus WT-LPS), q < 0.05 was considered as statistically significant. By comparisons of groups KO versus WT (i.e. KO-LPS vs. WT-LPS and KO + LPS vs. WT + LPS), differentially expressed genes passing p < 0.05 were considered as statistically significant. If setting the significance border to q < 0.05 only *Fcna* would be considered as differentially expressed between KO and WT groups. Hierarchical cluster analysis was performed and visualized using the Qlucore Omics Explorer™ software. All hierarchical clusters are build using average linkage and heat map was generated based on mean m = 0, variance 1 normalization.

### Statistics

All data, except microarray data, was analyzed using GraphPad Prism Software, version 6. Kaplan–Meier survival curves were compared using the log-rank test. Comparisons between groups of WT and KO mice were calculated using the unpaired Student’s t-test and p < 0.05 was considered statistically significant. Comparisons of serum cytokine levels were subjected to post hoc Bonferroni correction for multiple testing (22 comparisons).

## Electronic supplementary material

Supplementary information
