# Supplementary material for: Ficolins do not alter host immune responses to lipopolysaccharide-induced inflammation in vivo
Source: Sci Rep. 2017 Jun 20;7:3852. doi: 10.1038/s41598-017-04121-w (PMC5478672; doi:10.1038/s41598-017-04121-w)
Supplement: Supplementary file 1 — Supplementary information [file 41598_2017_4121_MOESM1_ESM.pdf]

Ficolins do not alter host immune responses to lipopolysaccharide-induced inflammation *in vivo*

Ninette Genster, Olga Østrup, Camilla Schjalm, Tom Eirik Mollnes, Jack B. Cowland and Peter Garred

Supplementary information

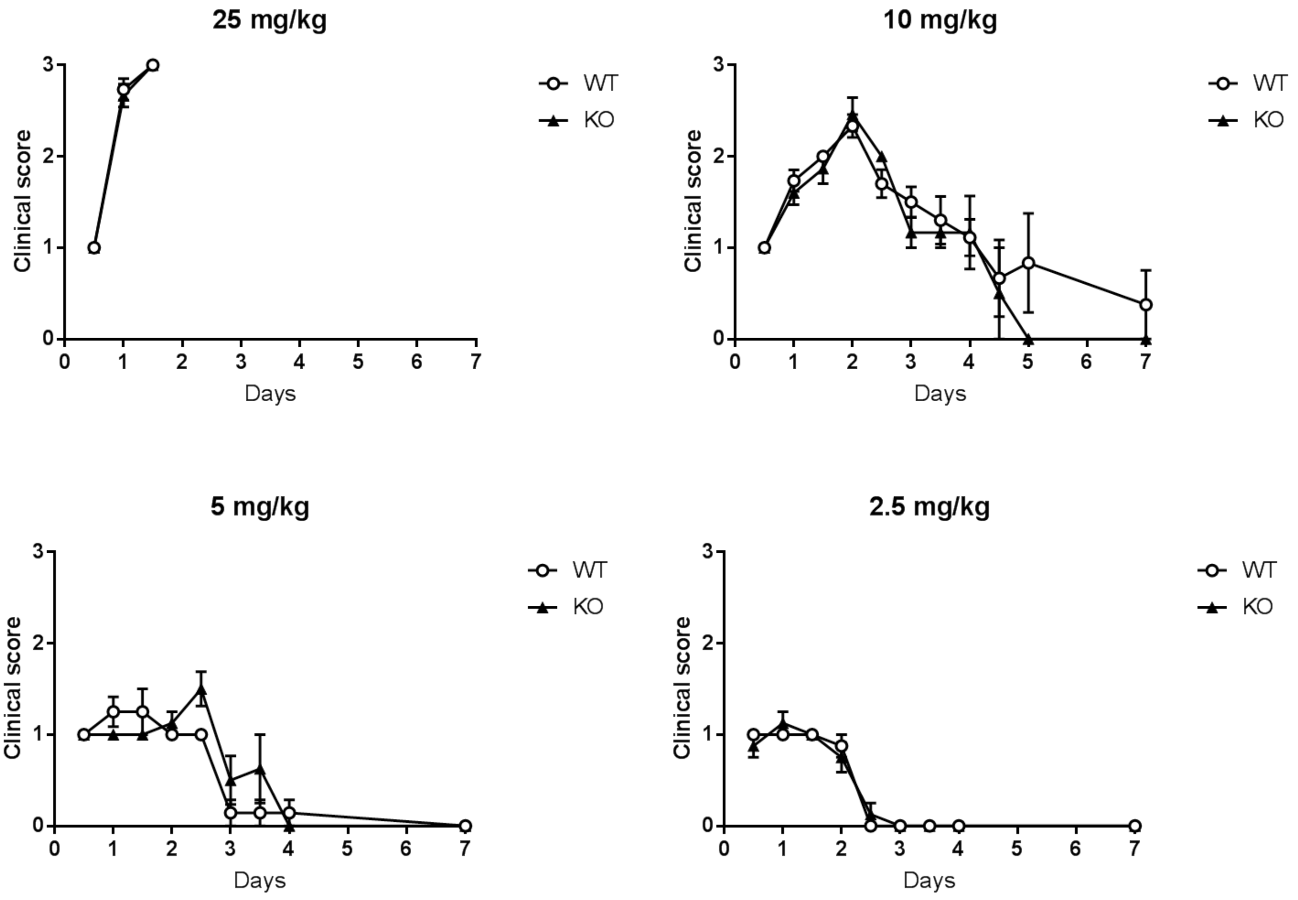

Supplementary Figure S1: Clinical scores are similar in ficolin KO mice and WT mice after LPS challenge. WT and ficolin KO mice were intraperitoneally injected with LPS of indicated doses and monitored for clinical signs of disease twice a day for seven days. The severity of the clinical appearance was scored as follows: 0, not affected; 1, slightly affected (mild piloerection and slightly hunched posture, normal activity); 2, affected (hunched posture, mild piloerection, less active); 3, severely affected (very hunched posture and severe piloerection, inactive). Animals were euthanized if they reached a score of 3. Results were obtained from one (5 mg/kg and 2.5 mg/kg; n=8) or two (25 mg/kg and 10 mg/kg; n=15) experiments.

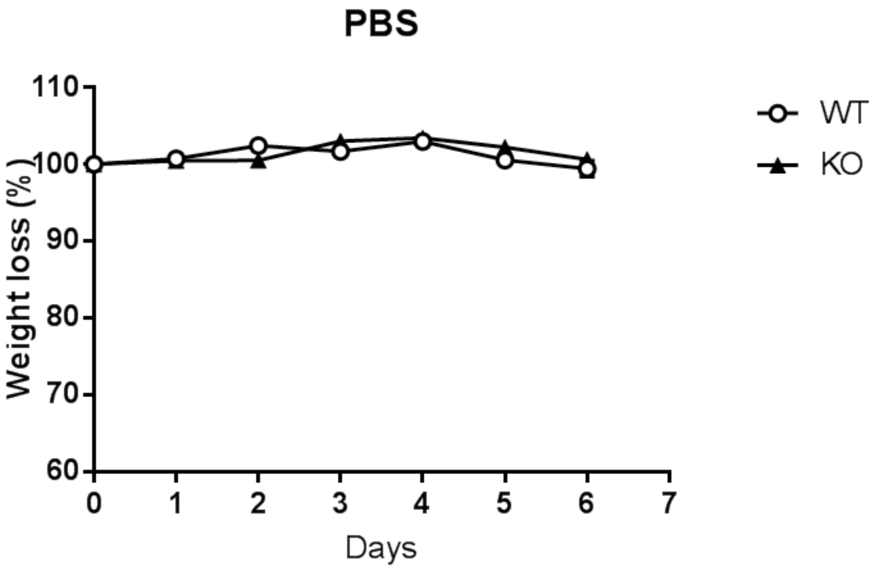

Supplementary Figure S2: Body weight change in control mice receiving PBS. Ficolin KO and WT mice received 100µl i.p. of PBS, and their body weight was recorded each morning for 7 days.

Supplementary Table S1: List of differentially expressed genes between naive KO and WT mice (cut-off: fold-change >1.5 and p< 0.05). Ranked by p-value.

**Gene Symbol**	**Gene title**	**p-value**	**q-value**	**Fold change**
Fcna	ficolin A	0.00012096	0.999820586	0.040523436
Reg2	regenerating islet-derived 2	0.001911474	0.999820586	6.95572138
Fndc9	fibronectin type III domain containing 9	0.006271631	0.999820586	0.657547295
Ifit1	interferon-induced protein with tetratricopeptide repeats 1	0.010425449	0.999820586	1.64212811
Olfr1057	olfactory receptor 1057	0.013770487	0.999820586	1.55121636
Cryaa	crystallin. alpha A	0.01669281	0.999820586	1.75098479
Nav3	neuron navigator 3	0.018182895	0.999820586	0.659860671
Platr16	pluripotency associated transcript 16	0.019339398	0.999820586	1.51354682
Igkv4-63	immunoglobulin kappa variable 4-63	0.019434186	0.999820586	1.80579424
Igkv6-14	immunoglobulin kappa variable 6-14	0.023451715	0.999820586	2.95447111
Tnfrsf8	tumor necrosis factor receptor superfamily. member 8	0.024352936	0.999820586	1.64436829
Nr1d1	nuclear receptor subfamily 1. group D. member 1	0.024693266	0.999820586	0.367080063
Cxcl10	chemokine (C-X-C motif) ligand 10	0.026220205	0.999820586	1.53251374
Tmem181c-ps	transmembrane protein 181C. pseudogene	0.027257524	0.999820586	0.652070522
Adgrg2	adhesion G protein-coupled receptor G2	0.029700616	0.999820586	0.657031059
Ighv2-7	immunoglobulin heavy variable 2-7	0.030730346	0.999820586	5.31560183
Ighv1-77	immunoglobulin heavy variable 1-77	0.031079341	0.999820586	0.555991113
Swsap1	SWIM type zinc finger 7 associated protein 1	0.031442849	0.999820586	0.580097198
Hist1h2ak	histone cluster 1. H2ak	0.035253284	0.999820586	1.63078511
Ighv1-47	immunoglobulin heavy variable 1-47	0.035945476	0.999820586	1.84727669
Npas2	neuronal PAS domain protein 2	0.037697815	0.999820586	1.5466001
Olfr372	olfactory receptor 372	0.043586437	0.999820586	1.6012516
Il22ra2	interleukin 22 receptor. alpha 2	0.046412171	0.999820586	0.601248503
Fpr2	formyl peptide receptor 2	0.049561675	0.999820586	1.64520431

Supplementary Table S2: List of differentially expressed genes between LPS-challenged KO and WT mice (cut-off: fold-change >1.5 and p< 0.05). Ranked by p-value.

Gene Symbol	Gene title	p-value	q-value	Fold change
Fcna	ficolin A	2.10E-06	0.06515653	0.077078208
Coq4	coenzyme Q4 homolog (yeast)	0.000182203	0.803396983	0.434642166
Fcamr	Fc receptor, IgA, IgM, high affinity	0.002163024	0.803396983	1.53612971
Zfp932	zinc finger protein 932	0.002660119	0.803396983	1.89745629
Ctrcos	chymotrypsin C (caldecrin), opposite strand	0.005476399	0.803396983	0.571846545
Ighv1-59	immunoglobulin heavy variable V1-59	0.006884177	0.803396983	0.445768505
Olfr1487	olfactory receptor 1487	0.008417016	0.804140389	1.69576168
Slc25a2	solute carrier family 25 (mitochondrial carrier, ornithine transporter) member 2	0.008797918	0.814551197	0.657744825
Nudt15	nudix (nucleoside diphosphate linked moiety X)-type motif 15	0.009738726	0.814551197	0.666325927
Cdh1	cadherin 1	0.009979862	0.814551197	0.640527904
Igkv11-125	immunoglobulin kappa variable 11-125	0.010825898	0.814551197	0.633507371
Rny1	RNA, Y1 small cytoplasmic, Ro-associated	0.011013516	0.814551197	0.553824246
Reg3a	regenerating islet-derived 3 alpha	0.011271817	0.816585191	0.134610847
LOC105246765	uncharacterized LOC105246765	0.011468274	0.816585191	0.624439299
Hist1h3e	histone cluster 1, H3e	0.011741141	0.816585191	0.57465446
Zfp595	zinc finger protein 595	0.0123264	0.828068311	1.5176791
Igkv3-10	immunoglobulin kappa variable 3-10	0.013670111	0.828068311	1.85223401
Pla2g1b	phospholipase A2, group IB, pancreas	0.014615277	0.828068311	0.10981901
Mir101c	microRNA 101c	0.015072551	0.828068311	1.91405642
Serpina3b	serine (or cysteine) peptidase inhibitor, clade A, member 3B	0.016384844	0.828068311	1.61582017
Prss3	protease, serine 3	0.016808209	0.828068311	0.622020245
Olfr584	olfactory receptor 584	0.017714295	0.830431148	1.92443252
Reg2	regenerating islet-derived 2	0.018995334	0.852992711	0.100611478
Krtap4-13	keratin associated protein 4-13	0.019402944	0.854114837	1.60959744
Igkv10-96	immunoglobulin kappa variable 10-96	0.019732783	0.855886334	1.72189844
n-R5s56	nuclear encoded rRNA 5S 56 [Source:MGI Symbol;Acc:MGI:4421901]	0.021673194	0.857472691	0.548083127
Igkv10-95	immunoglobulin kappa variable 10-95	0.021787262	0.857472691	1.6981535
Hist1h2bg	histone cluster 1, H2bg	0.024767719	0.861498752	0.509057283
n-R5s196	nuclear encoded rRNA 5S 196 [Source:MGI Symbol;Acc:MGI:4422061]	0.025796929	0.861498752	0.607967734
Hist1h4d	histone cluster 1, H4d	0.026686489	0.861498752	0.399555147
n-R5s151	nuclear encoded rRNA 5S 151 [Source:MGI Symbol;Acc:MGI:4422013]	0.028660288	0.861967799	0.613768578
Reg3g	regenerating islet-derived 3 gamma	0.029942118	0.861967799	0.154662997
Hist1h2ae	histone cluster 1, H2ae	0.031728078	0.861967799	0.555272102
Cep55	centrosomal protein 55	0.033315942	0.861967799	1.55351198
Fthl17b	ferritin, heavy polypeptide-like 17, member B	0.033333356	0.861967799	0.642588377
Ighv1-78	immunoglobulin heavy variable 1-78	0.03336336	0.861967799	2.43485093
Ighv1-36	immunoglobulin heavy variable 1-36	0.034780014	0.861967799	1.54845917
Pnliprp2	pancreatic lipase-related protein 2	0.036462845	0.861967799	0.111594163
Tff2	trefoil factor 2 (spasmolytic protein 1)	0.039169234	0.861967799	0.240793243
Reg3d	regenerating islet-derived 3 delta	0.039892065	0.861967799	0.155789137
Hist1h3d	histone cluster 1, H3d	0.040310591	0.861967799	0.54826051
Klk1b5	kallikrein 1-related peptidase b5	0.04062453	0.861967799	0.26835233

Igkv19-93	immunoglobulin kappa chain variable 19-93	0.041749687	0.863045545	1.64638305
Prss1	protease, serine 1 (trypsin 1)	0.042107912	0.863045545	0.232220396
Cuzd1	CUB and zona pellucida-like domains 1	0.042240663	0.863045545	0.173102692
Prss2	protease, serine 2	0.04342556	0.863045545	0.103432864
Zfp708	zinc finger protein 708	0.044107337	0.863045545	1.6364466
Serpini2	serine (or cysteine) peptidase inhibitor, clade I, member 2	0.044540078	0.863910481	0.620728016
Dmbt1	deleted in malignant brain tumors 1	0.045655552	0.865441403	0.116626412
Cpa2	carboxypeptidase A2, pancreatic	0.046108628	0.865441403	0.109841868
Rnase1	ribonuclease, RNase A family, 1 (pancreatic)	0.046442502	0.865441403	0.098636575
Igkv3-4	immunoglobulin kappa variable 3-4	0.048224008	0.865441403	2.5285902
Rps23	ribosomal protein S23	0.048446785	0.865441403	1.59689212
Tal1	T cell acute lymphocytic leukemia 1	0.048508227	0.865441403	0.659677744
n-R5s213	nuclear encoded rRNA 5S 213 [Source:MGI Symbol;Acc:MGI:4422078]	0.048617412	0.865441403	0.594864607
Trav3-4	T cell receptor alpha variable 3-4	0.048846953	0.865441403	0.62686789
Terc	telomerase RNA component	0.049255852	0.865441403	0.661127329
Snord13	small nucleolar RNA, C/D box 13	0.049383713	0.865441403	0.660013199
Sycn	syncollin	0.049563836	0.865441403	0.072664753
